# Climate-Friendly Seafood: The Potential for Emissions Reduction and Carbon Capture in Marine Aquaculture

**DOI:** 10.1093/biosci/biab126

**Published:** 2022-01-25

**Authors:** Alice R Jones, Heidi K Alleway, Dominic McAfee, Patrick Reis-Santos, Seth J Theuerkauf, Robert C Jones

**Affiliations:** University of Adelaide, Adelaide, South Australia, Australia; Nature Conservancy's Aquaculture Program, Arlington, Virginia, United States; University of Adelaide, Adelaide, South Australia, Australia; University of Adelaide, Adelaide, South Australia, Australia; NOAA National Marine Fisheries Office of Aquaculture, Silver Spring, Maryland, United States; Nature Conservancy's Aquaculture Program, Arlington, Virginia, United States

**Keywords:** climate change, carbon sequestration, food production, sustainability, blue carbon

## Abstract

Aquaculture is a critical food source for the world's growing population, producing 52% of the aquatic animal products consumed. Marine aquaculture (mariculture) generates 37.5% of this production and 97% of the world's seaweed harvest. Mariculture products may offer a climate-friendly, high-protein food source, because they often have lower greenhouse gas (GHG) emission footprints than do the equivalent products farmed on land. However, sustainable intensification of low-emissions mariculture is key to maintaining a low GHG footprint as production scales up to meet future demand. We examine the major GHG sources and carbon sinks associated with fed finfish, macroalgae and bivalve mariculture, and the factors influencing variability across sectors. We highlight knowledge gaps and provide recommendations for GHG emissions reductions and carbon storage, including accounting for interactions between mariculture operations and surrounding marine ecosystems. By linking the provision of maricultured products to GHG abatement opportunities, we can advance climate-friendly practices that generate sustainable environmental, social, and economic outcomes.

Food production systems operate within the resource-constrained biosphere and are often dependent on nonrenewable energy sources and increasingly compromised ecosystem services (Rasmussen et al. 2018). Because of this reliance on natural resources, climate change poses a tremendous challenge to the continued provision of nutritious, secure, and affordable food for the world's growing population (Porter et al. 2014, EAT-Lancet Commission 2019). However, food production also contributes significantly to climate change through both direct and indirect emissions of greenhouse gases (GHG; Springmann et al. 2018), estimated to account for 20% to 37% of anthropogenic GHG emissions annually (Poore and Nemecek 2018, Rosenzweig et al. 2020). There is a need to embed consistent GHG accounting practices into food production systems to effectively measure and move toward reducing emissions and climate change impacts (Godfray et al. 2010, IPCC 2019a). This will need to be a process of continual improvement within each food production sector, regardless of how its GHG footprint compares with other sectors.

Not all food is created equal in terms of climate impacts. Large variability exists in the GHGs emitted per portion of protein produced, both within and between food production sectors (Hilborn et al. 2018). The GHG emissions per unit of protein produced by aquaculture generally compare favorably with most livestock production and some wild-caught fisheries (Tilman and Clark 2014, MacLeod et al. 2020), but considerable variability exists within each food type (Poore and Nemecek 2018). Different GHGs have different global warming potential; therefore, they are often expressed as carbon dioxide equivalents (CO2e) so they can be compared. The best estimate of total annual GHG emissions from aquaculture (marine and freshwater) was 385 million metric tons (Mt) CO2e in 2008 (Hall et al. 2011). An updated estimate of 245 Mt of CO2e in 2017 was equivalent to approximately 0.49% of that year's total global anthropogenic GHG emissions, but this estimate is limited to emissions from shellfish, crustaceans and finfish (which, combined, account for approximately 93% of global aquaculture production; MacLeod et al. 2020). This is substantially lower than the GHG emissions footprint of terrestrial farming, estimated at 4 billion–6.6 billion metric tons (Gt) of CO2e per year (from agriculture and livestock combined; Smith et al. 2014). The lower emissions intensity of aquaculture is mostly attributable to a lack of direct GHG emissions from land-use change and more favorable feed conversion ratios (Hasan and Halwart 2009, Fry et al. 2018, MacLeod et al. 2019, MacLeod et al. 2020).

Although aquaculture's current contribution to GHG emissions from food production is small, there is high likelihood of aquaculture expanding, given the human health benefits and increasing social preferences for seafood (Clark et al. 2019). Therefore, it is critical to identify pathways to advance the growth of climate-friendly practices. Doing so provides an opportunity to avoid further environmental degradation associated with the expansion of food production (Ellis 2011). Ultimately, responsible development of aquaculture is a key strategy to meet growing food demand and nutritional needs and to achieve food security within planetary boundaries (EAT-Lancet Commission 2019, FOLU 2019, Stuchtey et al. 2020).

## Mariculture

**Figure 1.**
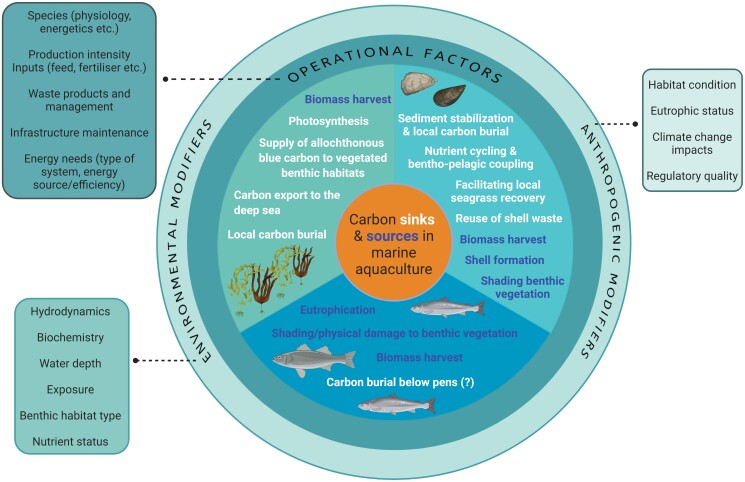
Potential carbon sources (the dark text) and sinks (the white text) associated with operational (on-farm) activities in the bivalve, seaweed and fed finfish mariculture sectors. Carbon sinks are parts of the farming process that lead to a net uptake of carbon from the environment, whereas sources are processes that lead to a net loss of carbon to the environment. The outer circles represent external factors that may influence carbon flow through mariculture farms or modify the magnitude of carbon sinks and sources. Sinks may not represent long term carbon sequestration, depending on the external influencing factors and the fate of the mariculture product.

In the present article, we examine the major sources of GHG emissions and assess both the opportunities for emissions reduction and the potential for carbon sequestration from three key marine aquaculture (mariculture) sectors: seaweed, bivalve, and fed finfish. We synthesize the available evidence on these sectors’ GHG footprint and explore the influence of farming practices on local marine carbon dynamics (figure 1, Box 1). The result is an improved understanding of the factors driving variability in GHG emissions footprint within and between these sectors. This enables us to provide guidance on climate-friendly mariculture practices that can reduce emissions or enhance marine carbon storage and to identify key knowledge gaps for future research.

**Box 1. Ocean carbon cycle.**

The oceans are a major driver of carbon cycling and the world's largest active carbon sink. Carbon in the oceans is either organic or inorganic and can be present in both dissolved and particulate forms. The main sources of carbon into the oceans are absorption of carbon dioxide from the atmosphere (resulting in an extremely large carbon sink of dissolved inorganic carbon) and organic carbon inputs, primarily from coastal runoff and riverine outflow. Dissolved inorganic carbon supports most marine food webs by enabling marine plants, from phytoplankton to giant kelp, to photosynthesise. Through this process, marine vegetation converts dissolved inorganic carbon to organic carbon, which is stored in their tissues and then either passed through marine food chains or buried in marine sediments where it may remain for hundreds to thousands of years (see box 2). Dissolved inorganic carbon can also precipitate to particulate inorganic carbon through calcification and the development of carbonate minerals, skeletons and shells—for example, in the formation of bivalve shells.

Cultivation of aquatic algae is dominated by the production of seaweeds in shallow to moderately deep coastal waters and, rarely, in offshore marine environments. Seaweeds are produced for both human and animal food as well as various nonfood products, such as carrageenan, agar, iodine, biofuels (biogas, biomethane) and fertilizers. Propagules (early life stages) are produced either in land-based hatcheries (largely for cultivation of temperate species) or via fragmentation of mature seaweed into seed stock on farms (more common for cultivation of tropical species), and then fixed to hanging longline systems, staked lines or floating rafts or racks to mature. Seaweed farming has more than tripled since 2000, representing 97% of the total 32.4 Mt of cultivated and wild-harvested seaweed produced globally in 2018 by 48 countries (FAO 2020), although a few countries in the Asian region dominate production (China, Indonesia, Republic of Korea, and the Philippines; FAO 2011–2021). Despite slower growth rates of global seaweed production in recent years, growth rates remain high in some nations (e.g., Indonesia), and an increasing number of countries are engaging with or indicating interest in this sector, including in temperate areas (FAO 2020). As nonfed organisms that can be readily grown in a range of conditions and locations, seaweed mariculture often has fewer environmental impacts than other types of plant or animal food production (Parodi et al. 2018).

Over 17.3 Mt of farmed shelled mollusks, mainly marine bivalves, were produced worldwide in 2018, accounting for 56.3% of the global production volume of marine and coastal aquaculture (FAO 2020). Global bivalve production (marine and freshwater) has more than tripled in the past 30 years because of expanding production in East Asia (FAO 2011–2021), especially in China (85% of global production in 2017; Wijsman et al. 2019, Willer and Aldridge 2020). Oysters account for approximately one third of mollusk production (33% in 2018), with clams, scallops and mussels accounting for a further 43% of production (FAO 2020). Most bivalves are farmed in sheltered, shallow near-shore or intertidal environments on raised infrastructure (e.g., long lines or racks; Forrest et al. 2009) or grown directly on the seabed in baskets or on loose shell seeded by natural recruitment (Dumbauld et al. 2009, Forrest et al. 2009). Because bivalves are low in the marine food chain, filter feeding on planktonic organisms, they do not require feed inputs except during breeding and rearing of larvae in hatcheries. Consequently, similar to seaweed cultivation, bivalve farming tends to have fewer environmental impacts than many other forms of food production (Parodi et al. 2018) and may provide positive ecological functions relevant to the health and resilience of marine environments (e.g., water clarification, nutrient cycling, biodeposition; Petersen et al. 2016, Rose et al. 2021). Understanding the extent to which different mariculture sectors may positively interact with surrounding ecosystems requires ongoing research. But there is growing evidence that some sectors and species will provide specific benefits. For instance, recent research highlighted the strong positive role of mussel farming on macrofaunal abundance (Theuerkauf et al. 2021).

Marine fed finfish are commonly farmed using coastal floating net pens and, to a lesser degree, land-based farming systems that use recirculating water (FAO 2020), with larval or juvenile stages supplied from hatcheries or caught in the wild (Halwart et al. 2007). Fed finfish production via mariculture is not yet a major contributor to total global aquaculture production (6.4% in 2018; FAO 2020), but the sector has comparatively large negative impacts on the marine environment (Volpe et al. 2013) and significant potential for future global expansion (Gentry et al. 2017a, Costello et al. 2020). China, Norway, and Indonesia are the top three producers of marine and coastal fed finfish mariculture, accounting for over 50% of global tonnage in 2018 (FAO 2020). Production is dominated by salmonids (FAO 2020)—in particular, Atlantic salmon (over a third of fed finfish mariculture in 2016; FAO 2018).

In the present article, we explore opportunities for these three mariculture sectors to support climate change mitigation through climate-friendly design and operational practices that can lead to either avoided emissions (reducing the quantity of GHGs emitted) or enhanced carbon sequestration (facilitating the uptake and storage of carbon, preventing its release to the atmosphere in the form of carbon dioxide or other GHGs such as methane). Although we have considered upstream (preproduction) and downstream (postproduction) activities, because they are important sources of emissions in the mariculture supply chain (table 1), we focus especially on identifying actionable, planning and operational changes that might provide opportunities for GHG abatement and sequestration during on-farm production (figure 1). As such, our assessment contributes to our understanding of designing and embedding sustainability in industrial practices, such as regenerative agriculture (Francis 2016, FOLU 2019), terrestrial agroecosystems (Power 2010) and ecoengineering (Strain et al. 2018).

**Table 1.** Major greenhouse gas emissions sources at different stages of the production cycle for the three key mariculture sectors.

Production stage	Source of emissions	Finfish	Bivalves	Seaweed
Upstream	Production and supply of eggs, larvae, or propagules	ü	∼	∼
	Terrestrial land-use change and degradation (e.g., for crops or livestock used in feed)	ü	û	û
	Feed production and processing (e.g., direct emissions from crop and livestock farms and wild caught fisheries)	ü	û	û
	Transport of feed to wholesale and mariculture operations	ü	û	û
On farm	Fuel use	ü	ü	ü
	Energy use	ü	ü	ü
	Infrastructure or maintenance	ü	ü	ü
	Coastal and subtidal land-use change and degradation	ü	∼	∼
	Nutrient or effluent impact and water treatment	ü	∼	∼
	Liquid oxygen and other chemicals used in production	ü	∼	∼
Downstream	Processing	ü	ü	ü
	Packaging and ice	ü	ü	ü
	Refrigeration	ü	ü	ü
	Transport	ü	ü	ü

*Note:* üand û indicate relevance to each sector, with ∼ showing where an emissions source is not typical when best practices are implemented, but may be relevant under some circumstances.

## Mariculture's greenhouse gas emissions footprint

GHG emissions from mariculture occur via many pathways, including upstream (e.g., finfish feed production), on-farm, and downstream (e.g., transportation) emissions (Gephart et al. 2016, Blanchard et al. 2017). Previous studies indicate that up or downstream activities contribute a considerable proportion of GHG emissions in mariculture, often more than on-farm operations, particularly when feed production is included as an upstream process (Pelletier and Tyedmers 2007, Volpe et al. 2010, Ziegler et al. 2012, Henriksson et al. 2013). Because downstream processes, such as transport throughout supply chains, can have a large impact on overall GHG emissions (Parker 2018), it can be difficult to generalize an emissions footprint to a sector or species level. Air transport has been shown to cause GHG emissions three to five times that of road freight, and 31 times greater than sea freight (Buchspies et al. 2011, Max et al. 2020). A specific example from Tamil Nadu (India) found that transport by ship, rail, or road increased the climate impact of maricultured seaweed by 14%, 51%, and 139% respectively, compared with the product's emissions footprint before leaving the farm (Ghosh et al. 2015). Therefore, downstream accounting heavily depends on where and how the product reaches the market.

**Figure 2.**
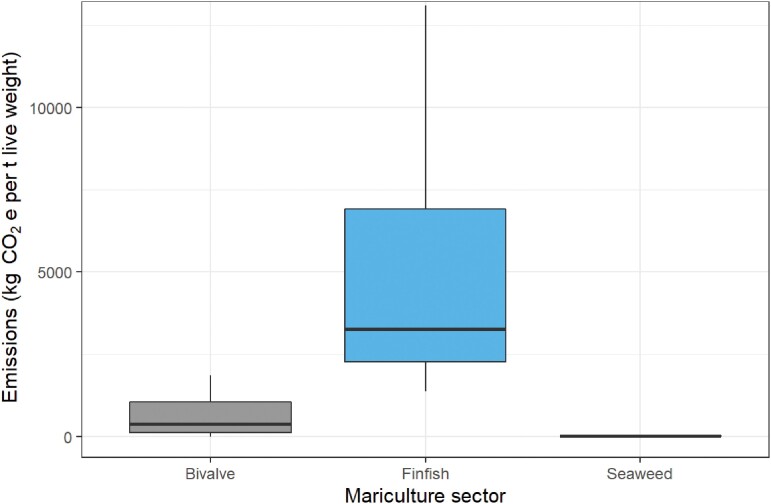
Comparison of total greenhouse gas emissions across all stages of the mariculture supply chain for bivalve, fed finfish and seaweed (excluding post harvest transport emissions). Emissions are reported in kilograms of carbon dioxide equivalents (CO_2_e) per metric ton live weight harvested. The bold horizontal line in each box shows the median value, the box extents are the upper and lower quartiles, whiskers extend to 1.5 times the interquartile range and outliers have been omitted from the plot (for ease of viewing). Data were collated through our literature search for each sector and are available in supplemental tables S1 and S3.

To evaluate trends in the major GHG emitting processes for the three sectors, we collated data from all the available literature, focusing on life cycle assessment (LCA) studies that contained quantitative and comparable GHG emissions data (fed finfish, 28; bivalves, 14; seaweed, 8). Details of the systematic review, including all reviewed literature, search terms, screening and eligibility criteria, and the values used in our assessment of GHG footprint are provided in supplemental tables S1 and S2 and the supplemental text. On the basis of these studies, we identified typical GHG emissions sources (table 1) and provided updated estimates of the total GHG emissions from each sector (excluding emissions from postharvest transport for the reasons discussed above; figure 2).

### Seaweed mariculture GHG footprint

Seaweed mariculture has lower reported GHG emissions than fed finfish and crustacean mariculture (Hall et al. 2011), although there are few LCAs for this sector (Froehlich et al. 2019, Halpern et al. 2019a), and they are biased toward temperate regions (table S1). This bias contrasts with the dominance of tropical seaweeds in global production. However, GHG emissions from on farm processes are likely to be even less in tropical areas because production typically involves limited infrastructure and mechanization (i.e., lower energy inputs) and is closer to shore (i.e., lower on farm transport and maintenance emissions). On the basis of the available data, total combined GHG emissions from upstream, on-farm and downstream processes (excluding postharvest transport) range from 11.4 to 28.2 kilograms (kg) of CO2e per metric ton of seaweed produced, with a median of 22 kg of CO2e (mean = 22.3 kg of CO2e; figure 2). Emissions from farming seaweed are lower and less variable (despite being based on fewer studies) than emissions from the bivalve sector and are considerably lower than emissions from fed finfish. However, including reported postharvest transport increases the maximum emissions estimate by an order of magnitude to 231 kg of CO2e per ton of seaweed (table S1).

With postfarm transport emissions excluded, the most emissions-intensive aspects of seaweed mariculture are usually on-farm activities, particularly electricity and fuel use, although there is variability across the studies in the activities included as a part of on-farm production (table 1; Hall et al. 2011, Taelman et al. 2015). In Nordic seaweed farms, upstream production of propagules combined with on-farm grow-out has been shown to account for 95% of total energy usage, with grow-out being the most energy demanding phase (Alvarado-Morales et al. 2013). In some temperate European mariculture systems, the infrastructure required to grow and dry seaweed on the farm (before transportation and processing) may account for almost 100% of the GHG emissions from the entire production process (van Oirschot et al. 2017; note that this study excluded hatchery seeding and processing). Consequently, although seaweed mariculture may represent a comparably low emissions production opportunity, attention should be given to sources of energy for cultivation, especially given efforts to move or expand seaweed cultivation to potentially energy intensive, offshore environments.

### Bivalve mariculture GHG footprint

As with seaweed, bivalve mariculture does not require feed inputs, which minimizes the associated land-based emissions from agricultural products. A recent estimate of emissions from bivalve production (11.1 tons of CO2e per ton of protein; Willer and Aldridge 2020) indicated emissions from this sector were just 7.6% of the average emissions from terrestrial (beef, pork, and chicken) protein production (Ray et al. 2019). Consequently, bivalve mariculture is increasingly discussed as a sustainable, climate-friendly source of nutrient-rich protein production for human consumption (Parodi et al. 2018, Willer and Aldridge 2020). Excluding postharvest transport, emissions estimates range widely, from –5 (i.e., bivalves were a net sink of carbon) to 1874 kg of CO2e per ton wet weight, with a median estimate of 392 kg of CO2e, well below the median of fed finfish but higher than seaweed (figure 2). The inclusion of postharvest transport increases the maximum emissions estimate to 2744 kg of CO2e per ton wet weight. The range of GHG emissions reported for this sector is, in part, due to the diverse production systems used in bivalve mariculture (table S1).

Few bivalve studies separate GHG emissions contributions by production stage (table S1). Those that have, suggest that on-farm energy and fuel usage contribute most to this sector's GHG footprint (Iribarren et al. 2010, Fry 2012), similar to seaweed mariculture. For example, on-farm operations contribute 60% and 79% of GHG emissions respectively to Scottish suspended mussel and intertidal oyster production, with farming materials (ropes, mesh bags, trestle tables) generating the remainder. The on-farm process of cleansing oysters before sale (depuration) can account for nearly a quarter of combined upstream and on-farm GHG emissions (310 kg of CO2e per ton wet weight; Fry 2012).

Importantly, bivalve shell formation is a net source of CO2 because, under most growth conditions, bivalves release more CO2 through respiration and the calcification process than the amount stored in their calcium carbonate shell (Jiang et al. 2014, Han et al. 2017). However, most LCA studies have not incorporated CO2 production from shell building into GHG emissions accounting. Including CO2 generated from shell formation increased mean emissions estimates by 219% (Ray et al. 2018; the data from this study are included in our assessment), emphasizing the critical importance of accurate GHG accounting, which incorporates biological and environmental processes.

### Fed finfish mariculture GHG footprint

**Figure 3.**
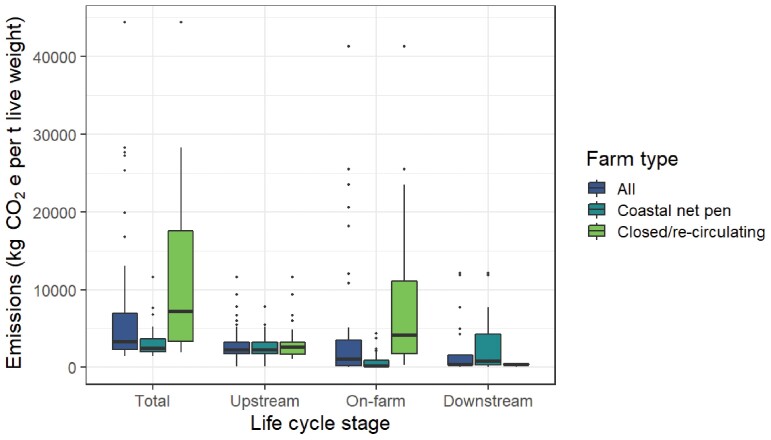
Summary of greenhouse gas emissions from fed finfish mariculture reported in kilograms of carbon dioxide equivalents (CO2e) per ton live weight harvested (excluding post harvest transport emissions). Data were collated from existing studies and, where possible, have been grouped into upstream, on-farm and downstream activities and separated by production system type (all systems pooled, closed or recirculating systems and open net pen systems). The bold horizontal line in each box shows the median value, the box extents are the upper and lower quartiles, whiskers extend to 1.5 times the interquartile range and outliers are shown by black points.

Our median estimate of total GHG emissions for fed finfish across the supply chain (excluding postfarm transport) is 3271 kg of CO2e per ton wet weight, far greater than that of seaweed or bivalve culture (figure 2). However, there is large variability in total emissions estimates (1382–44,400 kg of CO2e per ton wet weight; figure 3, table S1), which vary among species, the source and composition of feed, geographic locations, local or national energy sources (e.g., low-carbon or fossil fuels), but mainly by farming system (coastal net pens versus closed or recirculating systems; figure 3). For example, Atlantic salmon can be produced with relatively low GHG emissions comparable to or only marginally higher than some mollusks (Pelletier et al. 2009, Hilborn et al. 2018), but such low-emissions production is rarely reported in the LCA literature (table S1).

The larger GHG footprint of fed finfish (figure 2) is commonly attributed to the emissions intensity of feed supply (Hilborn et al. 2018, Parodi et al. 2018, MacLeod et al. 2020), and is similar in net pen and RAS farming (figure 3). Emissions from feed supply include crop agriculture and associated land-use change, wild-caught fish meal or oil, as well as feed processing and transport to farms (table 1; e.g., Ellingsen and Aanondsen 2006, Pelletier and Tyedmers 2007, Iribarren et al. 2010, Parker 2018, MacLeod et al. 2019). As fed species mariculture is growing faster than nonfed species (FAO 2020), there is an urgent need to tackle the emissions intensity of feed production and the complexity in supply chains that leads to a greater GHG footprint (Asche et al. 2018).

On-farm GHG emissions are relatively high for all finfish production systems (median = 1040 kg of CO2e per ton wet weight), especially if compared with the total GHG footprint of seaweed or bivalve farming (figure 2). On-farm emissions are greatest in closed or recirculating systems (figure 3), which are located onshore and require high energy usage for pumping, filtering, and maintaining water temperature and oxygen levels (Aubin et al. 2009, Ayer and Tyedmers 2009, Ahmed and Turchini 2021). However, the most common fed finfish mariculture system globally is a nonintegrated (i.e., monoculture), anchored net pen system, operating over soft sediment seafloors in coastal waters less than 30 meters (m) deep (Halwart et al. 2007, Ayer and Tyedmers 2009, FAO 2020), within 3 nm of the coast and not offshore or in the open ocean (Froehlich et al. 2017). In coastal net pen farms, upstream and downstream activities typically contribute more GHG emissions than on-farm operations (figure 3), with fuel, energy use, infrastructure construction and maintenance responsible for most on-farm emissions (figure 3, table S1; Ellingsen and Aanondsen 2006, Hall et al. 2011, Ziegler et al. 2012). However, few LCA studies consider environmental emissions for net–pen systems during the on-farm stage (although these may be considerable; Hu et al. 2012).

Environmental emissions from fed finfish mariculture are released via two main pathways: GHGs released from the decomposition of waste food and nutrient-enriched seawater (e.g., methane, nitrous oxide) and carbon emissions from degraded seafloor habitats that are similar to terrestrial agriculture's climate impact through emissions from land-use change (Hall et al. 2011, Flynn et al. 2012, IPCC 2019b). Locating net pens in shallow, low energy coastal areas is preferred for increased accessibility and protection from damaging waves (Trujillo et al. 2012, Kapetsky et al. 2013). However, this means that farms are tightly linked with the surrounding marine environment through water and waste exchange, which increases the likelihood of degrading seafloor habitats (Halwart et al. 2007, Volpe et al. 2013, Abdou et al. 2018) and the subsequent release of environmental emissions.

Feed for finfish mariculture is high in nitrogen, and up to 95% (range of 1%–95%) may be lost as waste to the surrounding marine environment (Findlay and Watling 1994, Price et al. 2015). The expansion and intensification of fed finfish mariculture (FAO 2020) have considerably increased the cumulative nutrient load and subsequent eutrophication in coastal marine environments (Volpe et al. 2013, FAO 2020). However, unfortunately, we lack consistent and comparable data with which to better understand the influence of farming intensity or stocking densities. Increased nitrogen and particulates in the water (which contribute to turbidity) can lead to the loss or degradation of seagrass habitats below and adjacent to the farms (Thomsen et al. 2020). This can result in GHG emissions through release of stored blue carbon in the plants and sediments below them (see Box 2) and can reduce the capacity for future blue carbon sequestration (Jiang et al. 2018, Salinas et al. 2020). Nutrient enrichment of sediments can also increase microbial activity, accelerating the sediment carbon cycle and further release of stored blue carbon (Liu et al. 2017), as well as other potent GHGs, such as methane (Chen et al. 2016) and nitrous oxide (Hu et al. 2012).

**Box 2. Blue carbon.**

Vegetated coastal marine habitats, specifically seagrasses, mangroves and tidal marshes, contain up to 50% of all the organic carbon buried in ocean sediments (Duarte et al. 2005). These are commonly referred to as blue carbon ecosystems and they can accumulate and store greater carbon volumes at faster rates than terrestrial forests, because of their ability to trap and bury organic matter (Mcleod et al. 2011). Their waterlogged soils tend to be low in oxygen (anoxic), decreasing the chance that stored organic carbon becomes oxidized and released as CO_2_ unless disturbed (Pendleton et al. 2012, Marbà et al. 2015, Sasmito et al. 2019). The effect of salinity on soil chemistry leads to minimal methane releases, despite the soils being anoxic (Kroeger et al. 2017, Rosentreter et al. 2018). Moreover, blue carbon ecosystems have the capacity to accrete soil rapidly to keep up with changing sea levels and maintain their preferred positions in the coastal zone (ensuring adequate light and tidal exposure; Rogers et al. 2019). All these traits make them some of the most significant biological carbon sinks in the world (Duarte et al. 2013). Unfortunately, blue carbon habitats are often degraded by human activities (Halpern et al. 2019b). Global mangrove decline due to deforestation and coastal land-use change was estimated at 35% of their total area up to 1999, with ongoing losses estimated between 0.2% and 8% per year (Friess et al. 2019). In particular, large areas of mangroves have been cleared to make way for coastal mariculture, (Kauffman et al. 2017), which leads to GHG emissions (Bulmer et al. 2017) and can halve sediment organic carbon stocks compared with intact mangroves (Kauffman et al. 2018, Arifanti et al. 2019). Mangrove and tidal marsh conversion for mariculture in China alone is estimated to emit 15–82 million tons of CO_2_e per year (Wu et al. 2020). Over 29% of the world's seagrass area has also been lost since 1879, with ongoing annual losses estimated at up to 7% (Waycott et al. 2009). Global seagrass loss may cause annual blue carbon emissions of 0.15 billion–1.02 billion tons of CO_2_e (Pendleton et al. 2012). Coastal mariculture operations can impact seagrass ecosystems through direct disturbance or shading, leading to losses of up to half the sediment carbon stock (Lovelock et al. 2017, Trevathan-Tackett et al. 2018). In addition, localized nutrient enrichment commonly associated with fed finfish mariculture causes metabolic stress and reduced seagrass growth (de Kock et al. 2020), poor recruitment (Díaz-Almela et al. 2008), and, ultimately, seagrass loss around pens (e.g., Delgado et al. 1999, Ruiz et al. 2001, Herbeck et al. 2014, Cullain et al. 2018, Thomsen et al. 2020), leading to decreases in both stored blue carbon and ongoing sequestration potential (Apostolaki et al. 2011, Jiang et al. 2018, Liu et al. 2020).

Although the total global area of mariculture's built infrastructure footprint was recently estimated to be at least 23,000 square kilometers (Bugnot et al. 2021), unfortunately, there are no georeferenced global data available on the spatial distribution of active fed finfish net pens, nor their overlap with seagrass habitats. However, this overlap may be considerable, because seagrass generally thrives in the same shallow, protected areas that suit net pen mariculture (Short et al. 2007). We estimate that the release of environmental emissions resulting from seagrass degradation and associated blue carbon stock losses could conservatively equate to 4.1%–16.3% of the total global aquaculture emissions in 2017, depending on the carbon stock loss scenario and assumed proportion of overlap between farms and seagrasses (see the worked example in supplemental file S2 and table S4). There will also be associated losses of future blue carbon sequestration potential, which may be up to 0.31% of aquaculture's GHG footprint per year (file S2 and table S4). None of the published LCA studies accounted for these marine environmental emissions in the on-farm part of the fed finfish life cycle. Neither were there data on the land-based footprint of closed or recirculating systems or their impact on terrestrial environment emissions through land-use change, although most studies included similar types of environmental emissions associated with terrestrial ecosystem degradation and land-use change due to farming of finfish feed ingredients. This omission is likely due to significant knowledge gaps around the drivers and potential magnitude of environmental emissions from mariculture-related degradation (Hall et al. 2011, Abdou et al. 2017).

## Opportunities for climate-friendly mariculture

There is potential for climate-friendly design and operation to improve the GHG footprint of seaweed, bivalve, and fed finfish mariculture. Opportunities to reduce emissions come through approaches that lead to direct emissions reduction, actions that reduce indirect (e.g., environmental) emissions (figure 1), farming approaches that enhance carbon sequestration, and product uses that offset GHG emissions from other sources (i.e., agriculture and livestock) using mariculture products. These opportunities are explored in more detail below.

### Reducing direct and indirect GHG emissions

We have identified some common approaches to reducing emissions from on-farm energy and fuel use across all three sectors. These include shifting to low-emissions energy sources and biofuels, as well as sustainable building materials where available (Myrvang 2006, Aubin et al. 2009, Mungkung et al. 2014). Unless low-emissions alternatives to fossil fuels can be readily adopted, the potential socioecological benefits of large, offshore mariculture development (Gentry et al. 2017b) could be diminished by a need to increase fuel use to enable distant production at scale. In fed finfish mariculture, changing from diesel oil to natural gas (a lower emissions fuel) has been shown to reduce nitrous oxide (a potent GHG) emissions from farmed salmon by 85% and CO2 emissions by 20% (Ellingsen and Aanondsen 2006). In addition, multiple studies identify that the reuse of materials (rafts, ropes, etc.), and the specific energy sources used have significant potential to reduce emissions (e.g., Langlois et al. 2012, Jung et al. 2016). For example, emissions from on-farm energy use are four times lower using nuclear than coal generated electricity (Aubin et al. 2009). Consideration of on-farm energy requirements and supply sources are particularly relevant when establishing large-scale farming sites, especially those located offshore (e.g., Taelman et al. 2015). The colocation of offshore mariculture farms with energy generation (e.g., windfarms; Buck and Langan 2017), increased use of low-emissions energy supplies, and the on-site use of seaweed-derived biofuel products for energy production (Aitken et al. 2014) will be critical to achieving sustainable expansion. However, changes in a country's energy portfolio and the market forces driving the availability and affordability of biofuels are likely to occur at a national or regional level, with single farm operators having little control over these overarching drivers of on-farm GHG emissions (Hall et al. 2011). Therefore, we focus the rest of this overview article on providing recommendations for readily actionable changes to the operation and design of on-farm activities.

### Opportunities for avoided emissions in finfish mariculture

**Figure 4.**
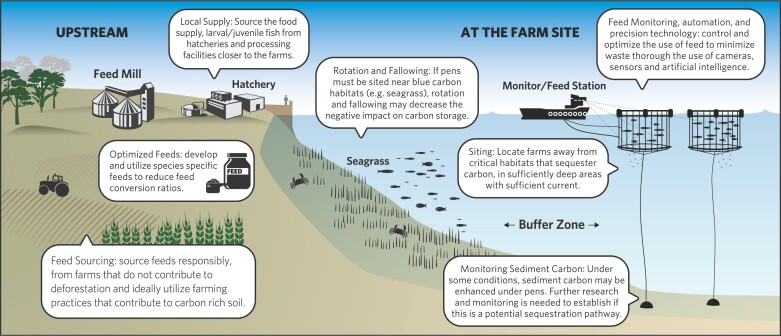
Potential pathways for greenhouse gas emissions reductions from fed finfish mariculture.

Site selection for coastal fed finfish mariculture should exclude areas of seagrass and other sensitive blue carbon habitats where possible (figure 4), although complete avoidance may not be practical in some regions, due the widespread distribution and seasonal variations in the presence and density of seagrasses. Moving net pens into deeper coastal water (e.g., less than 30 m) helps reduce overlap with seagrass habitats, which are more common in shallower water with greater light penetration (figure 4). However, reducing blue carbon emissions through a shift to deeper water will need to be traded off against the potential for increased operational GHG emissions from fuel use and maintenance, as well as the need for more robust farm infrastructure in offshore conditions (Holmer 2010). Similarly, moving production to onshore closed or recirculating systems can reduce impacts on local marine environments. However, onshore systems have considerably higher emissions from operational energy use (figure 3, table S1), and the disposal of waste water into coastal seas (even though it is often treated) and sludge effluents on land can still result in environmental GHG emissions. Better accounting for environmental emissions from mariculture is crucial to effectively compare GHG footprints across scales and geographies and to monitor the climate costs and benefits of moving production offshore or into closed or recirculating onshore systems against coastal net pen operations.

Where seagrass avoidance is not possible when siting coastal net pens, reduction in environmental GHG emissions may be achieved through coastal farming practices such as fallowing or regularly shifting the location of infrastructure within the broader farm area (figure 4; Lauer et al. 2009, Kletou et al. 2018). Although, even with fallowing, chronic nutrient enrichment of sediment and water and the associated potential for environmental emissions of stored blue carbon may persist (Carroll et al. 2003, Díaz-Almela et al. 2008, Thomsen et al. 2020). In any case, actions that reduce the exposure of seagrasses to negative impacts are preferable to ecosystem recovery attempts after damage has occurred. If seagrass meadows are lost, recovery can take decades and may not occur at all if mariculture activities remain or if the environment has changed significantly since seagrass loss (Tanner et al. 2014). Regulated baseline surveys (before farm installation) and ongoing monitoring of seagrass condition (e.g., benthic videos) along with water quality monitoring can help minimize harm to seagrasses (EPA 2020 2021), reducing the potential for environmental GHG releases from seagrass blue carbon stores.

Leveraging marine environmental modifiers such as local hydrodynamics when choosing fed finfish mariculture sites can help to reduce eutrophication-related environmental GHG releases (figures 1 and 4; Price et al. 2015). Siting of pens in areas with stronger currents and low water residence times (high flushing rates) reduces the concentration of nutrients and accumulation of particulates on the seafloor (such as uneaten food and feces; Middleton and Doubell 2014, Duman et al. 2020). However, stronger tides, currents, and wave action can also pose a risk to farm infrastructure and moorings (Cromey et al. 2002, Bravo and Grant 2018) and can shift the spatial impact of eutrophication to downstream areas (Henderson et al. 2001). Carefully designed distribution of fed finfish stock within a farm site, on the basis of knowledge of local marine environmental conditions (e.g., flow speed and water depth), can reduce both extreme and cumulative impacts on the seabed, potentially being more important than stocking density alone (Burić et al. 2020). However, it is difficult to produce generic guidelines on depths, currents, and tidal conditions that reduce impacts while optimizing yield, because this is strongly affected by the species farmed, feeding practices, local marine environmental conditions, stocking densities, and the spatial arrangement of pens (Ali et al. 2011, Cardia and Lovatelli 2015, Doubell et al. 2015, Abdou et al. 2018). Evidence from pilot studies shows potential for nutrient removal through farming kelp (seaweed) next to salmon pens, which removes excess nitrogen inputs while also boosting seaweed growth rates and harvestable biomass (Wang et al. 2014). This nutrient removal strategy may indirectly reduce environmental GHG emissions caused by eutrophication from the salmon farm. However, there are issues of scaling, with each additional hectare of seaweed cultivation absorbing less than 0.5% of the excess nutrients introduced from the salmon farm (Fossberg et al. 2018).

Environmental GHG emissions from fed finfish mariculture may be decreased by shifting to species that require less feed or by altering the composition of feed, reducing eutrophication (Ellingsen and Aanondsen 2006, Han et al. 2018, Little et al. 2018, MacLeod et al. 2019). Improvements in feed conversion ratio (FCR, the amount of feed required for each kilogram of fish produced) for fed finfish species can be achieved through genetics (either selective breeding or genetic modification; Besson et al. 2016) and innovation in feed composition (Parker 2018, Hua et al. 2019). These actions can lead to improved protein transfer efficiency and reductions in food waste to the environment (Ballester-Moltó et al. 2017), as well as reducing the total volume of food required and the GHG emissions directly associated with feed production (Hall et al. 2011, MacLeod et al. 2020, Maiolo et al. 2020).

Implementing practices to avoid overfeeding reduces particulates and nutrient waste lost to the marine environment (Alongi et al. 2009, Besson et al. 2016, Ballester-Moltó et al. 2017), decreasing the direct and indirect (i.e., environmental) GHG emissions while boosting economic efficiencies. This can be achieved using advanced on-farm feed delivery systems (e.g., behavior-based automated, precision feeding systems; figure 3; Føre et al. 2018) Such technologies may not currently be within reach for operators in some developing countries, although early adoption in other countries may help drive down costs and increase accessibility in the longer term (Gentry et al. 2019). Mariculture may have greater scope for adopting technological innovation than other food production sectors, because it is a comparably young industry (Waite et al. 2014). There is also the potential to use seafood sustainability certification schemes to leverage improvements in feed and fish production systems that lead to GHG abatement (Madin and Macreadie 2015), particularly associated with the sourcing of sustainable feed ingredients (figure 4; Mariojouls et al. 2019).

### Opportunities for avoided emissions and emissions offsets in bivalve mariculture

**Figure 5.**
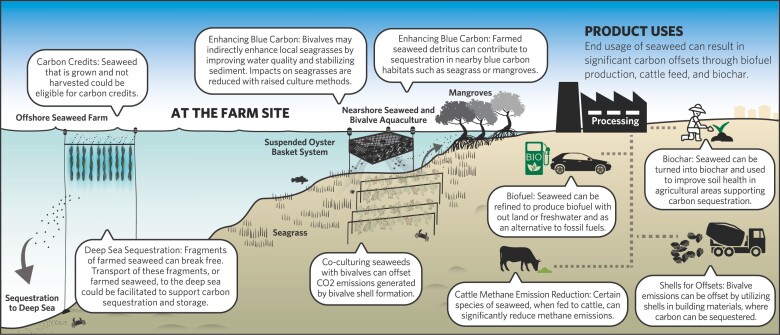
Potential pathways for greenhouse gas offsets, carbon storage and sequestration from seaweed and bivalve mariculture.

Bivalve culture in shallow waters regularly occurs in close proximity to or directly intermingled with seagrass meadows. Therefore, seabed disturbance and seagrass loss can occur when bivalve operations are established (Tallis et al. 2009), although estimates of the resulting environmental GHG emissions are not available as these are not accounted for in LCAs (table S1). Erecting poles or racks for raised farming can physically disturb seagrass, whereas ongoing shading beneath raised cultures reduces seagrass density and percentage cover (though empirical evidence for this is limited; Forrest et al. 2009). Nevertheless, raised bivalve culture appears to have fewer impacts on seagrasses than on-bottom culture, which displaces seagrass through disturbance and direct competition for space (Ferriss et al. 2019). Importantly, the structures used for bivalve mariculture do not necessarily preclude healthy seagrass meadows (Crawford et al. 2003, Dumbauld and McCoy 2015) and have been shown to stabilize sediment, improve benthopelagic nutrient transfer and boost neighboring seagrass growth under certain conditions (figure 5; Ferriss et al. 2019).

The method used to harvest mature bivalves has profound impacts on local benthic disturbance, seagrass cover and, therefore, blue carbon burial and storage. The mechanical dredging of on-bottom bivalve cultures disturbs seagrass beds and may hinder their recovery (Tallis et al. 2009, Dumbauld and McCoy 2015). Manual harvesting of raised mariculture (suspended cultures retrieved by hand; figure 5) is the practice least likely to disturb seagrass and buried carbon. Raised culture also avoids the direct competition with seagrass for space and reduces sediment resuspension compared with on-bottom culture. This both stabilizes sediment to allow seagrass recruitment and enhances or prolongs carbon storage by reducing oxidation of subsurface sediments (Forrest et al. 2009).

The volume of valuable, carbon-rich shell waste from bivalve aquaculture is considerable, estimated at up to 11.9 Mt per year (based on shells accounting for an average of 68.6% of total bivalve weight; Tokeshi et al. 2000). Shells can be turned into calcium carbonate (CaCO3) or calcium oxide (CaO), providing an abundant, cheap, and sustainable resource with broad industry application (FAO 2020), reflected by the market value of shell waste (between US$538 and $1783 per ton; Morris et al. 2019, van der Schatte Olivier et al. 2020). Unfortunately, given their potential value, most bivalve shells are discarded in landfills (10 Mt per year in China alone; Yao et al. 2014), where they eventually release the stored carbon to the environment, and carry a high disposal cost for the farmer (Yan and Chen 2015). This is despite calcium carbonate from limestone being mined in enormous quantities worldwide, primarily for cement production, at significant environmental cost (Morris et al. 2019). Repurposing shell waste as construction aggregate or for mortar mixes could potentially lead to long-term carbon storage while offsetting emissions from energy intensive, nonrenewable resources (figure 5). Markets already exist worldwide for whole and crushed bivalve shells as aggregate in construction (e.g., wall and road construction; Morris et al. 2019), and their use in cement production does not compromise performance (Yoon et al. 2004, Kumar et al. 2016), although shell availability may limit large-scale application. Other destructive uses of shell waste (e.g., calcium supplement in poultry grit, agricultural lime release) typically cause the release of the stored carbon (van der Schatte Olivier et al. 2020). Even so, the repurposed shell still offers a less emissions intensive alternative to mined CaCO3. Furthermore, under certain conditions, pulverized shell applied as liming agent may mitigate GHG emissions from agricultural soils and could therefore be considered an emissions offset (Hamilton et al. 2007). Oyster shell is also highly prized for oyster reef restoration as it provides an optimal settlement substrate (McAfee and Connell 2020). Although returning bivalve shells to the marine environment will eventually release the stored carbon as shells dissolve, there are considerable positive benefits of bivalve reef restoration, including indirect carbon sequestration through enhancing blue carbon habitats (Fodrie et al. 2017, Chowdhury et al. 2019, McAfee et al. 2020).

### Opportunities for avoided emissions and emissions offsets in seaweed mariculture

Although biomass yields from seaweed mariculture can be very high, often greater than those from terrestrial crops (Hughes et al. 2012), variability in the marine environment around farm sites affects productivity (i.e., growth rates and total biomass yields). This, in turn, can significantly affect production efficiencies and GHG emissions (Froehlich et al. 2019), as well as the potential for negative interactions with the marine environment (Campbell et al. 2019). Productivity can be enhanced through careful site and species selection or scaling up operations to optimize efficiency (Pechsiri et al. 2016, Seghetta et al. 2016), offering the potential for climate-friendly farm designs, farm siting, and species choices to support the delivery of GHG emissions reductions outcomes (figure 5).

Emerging markets for climate-friendly, nonfood seaweed bio products (such as biofuels and biochar) provide an opportunity to realize GHG emissions offsets from seaweed mariculture (figure 5; Seghetta et al. 2016, Seghetta et al. 2017, Laurens et al. 2020), although the magnitude of any benefit is dependent on the energy requirements and energy sources used in product processing. The upper limit for CO2 uptake through seaweed production is estimated at 2.48 Mt of CO2e (680,000 tons of organic carbon) per year. This equates to each square kilometer of cultivated seaweed having the potential to reduce fossil fuel emissions by approximately 1500 tons of CO2e a year (based on 2014 production quantities) if the biomass produced was diverted to the creation of biofuels (Duarte et al. 2017). Another innovative use of farmed seaweed is future feed, animal feed products (fish and livestock) that can achieve a net reduction in GHG emissions compared with current feed sources or provide a functional food value (improve fish health and, therefore, efficiency in production; e.g., Hua et al. 2019; and ruminant methane reduction; e.g., Kinley et al. 2020). However, there are few seaweed species suitable for these applications and they each have specific culturing requirements. The only species currently shown to deliver methane reduction when used in livestock feed is Asparagopsis taxiformis (Roque et al. 2021), a tropical species that is challenging to grow and may prove difficult to culture at the scales needed to generate significant GHG reductions. The production of seaweed-based biochar for use as a soil improver can also indirectly support climate change mitigation and offsets through agricultural soil improvement, because it contains recalcitrant carbon that facilitates long-term soil carbon sequestration (Roberts et al. 2015, Smith 2016). However, biochar is currently energy intensive to produce. Other barriers to the realization of consistent and scalable emissions reductions and offsets through seaweed bio products include the limited number of cultivated seaweed species and production environments, and technological and engineering constraints that reduce economic viability (Laurens et al. 2020). Should these barriers be overcome, there is great scope for future spatial expansion of this sector (Froehlich et al. 2019), which could support sustainable intensification and climate-friendly production.

### Carbon sequestration

There has been a rapidly increasing interest in mariculture's potential to support climate change mitigation via direct carbon storage and sequestration, especially through seaweed farming (Froehlich et al. 2019, Oceans 2050 2021) and bivalve farming (Hickey 2008). In this context, carbon sequestration is the direct movement of organic carbon in seaweed and bivalve biomass into long-term marine carbon stores, such as the deep ocean.

### Potential for carbon sequestration through seaweed mariculture

In natural environments, seaweed plays an essential and major role in ocean carbon regulation and carbon flux (box 1; Queirós et al. 2019). Natural seaweed habitats are typically associated with hard substrates, as opposed to soft sediment and, therefore, have lower inherent potential for direct transfer and sequestration of carbon to the sediment than blue carbon habitats (box 2). However, naturally growing seaweeds do donate organic carbon in the form of detritus to nearby receiver blue carbon habitats, where the material is trapped and buried in the sediment (Chung et al. 2011, Hill et al. 2015, Trevathan-Tackett et al. 2015). Net sequestration of particulate organic carbon from natural seaweed beds is estimated to equate to 4%–5% of the blue carbon sequestered into some mangroves (Queirós et al. 2019). Receiver habitats are also not exclusively shallow, vegetated blue carbon ecosystems; in fact, offshore export of seaweed detritus to deep (less than 1000 m) ocean sediments may account for approximately 90% of carbon sequestration from natural seaweed biomass (Krause-Jensen and Duarte 2016).

The transfer of organic carbon to receiver habitats (both deep sea and shallow blue carbon environments) can also occur from seaweed mariculture farms, although there are many unknowns around this process including the magnitude, consistency, and potential benefits or negative impacts. This process occurs through detachment of seaweed from lines, breaking off of fronds, or erosion and decay of cultivated plants (figure 5; Hyndes et al. 2012, Hill et al. 2015). Carbon from maricultured seaweed may also be moved indirectly into nearby coastal sediments through grazing organisms that consume biomass at the farm and move into neighboring marine ecosystems, although, again, the magnitude of this transfer and its ultimate impact on carbon sequestration are currently unknown (Sondak et al. 2017). Generally, seaweed losses could be considered a small proportion of the total farmed biomass (approximately 4.5%), because this transfer is minimized through operational practices (e.g., siting choice, orientation and maintenance) that aim to improve production efficiency (Campbell et al. 2019). Also, in some jurisdictions, there are guidelines or regulations around seaweed farming that require siting away from sensitive habitats, such as seagrass and rocky reefs (e.g., Eklof et al. 2006). Although this requirement may serve a useful purpose in reducing the risk to marine habitats from farm shading, disturbance, introduced species, farming infrastructure, or operations and waste discharges, it may also reduce the opportunity for seaweed farms to act as blue carbon donors. To establish seaweed farming as an effective blue carbon donor, we need to better understand the rate of carbon transfer from farms to nearby receiver habitats, and the environmental modifiers (e.g., flow rates and exposure; figure 1) that influence this connectivity (figure 5). Alongside efforts to design seaweed farms that enhance blue carbon donation, there would need to be careful monitoring of the potential benefits and the magnitude of carbon sequestration achievable, as well as the potential for negative impacts from movement of biomass to blue carbon habitats, such as shading or smothering.

The Intergovernmental Panel on Climate Change (IPCC) has recommended macroalgal production as a research area for climate change mitigation (IPCC 2019a), an ocean-based climate change mitigation also suggested by the High-Level Panel for a Sustainable Ocean Economy (Hoegh-Guldberg et al. 2019, Stuchtey et al. 2020). Although seaweed is a low-emissions mariculture sector (figure 2) that stores large amounts of carbon in the farmed plant biomass, once the product is harvested, carbon is either released or transferred up the food chain. Therefore, this sector should not, by default, be considered a source of long-term carbon sequestration (Duarte et al. 2017).

The intentional farming of seaweed as a means to capture and sequester anthropogenic CO2 could function in a similar way to carbon farming initiatives on land (Froehlich et al. 2019, Hoegh-Guldberg et al. 2019). This approach would rely on a nonharvest mariculture model, where biomass is either retained *in situ* or allowed (or facilitated) to sink to the deep sea (less than 1000 m), where the carbon can be sequestered for long periods of time (figure 5; Froehlich et al. 2019). Noting that passive sinking requires less energy and labor compared with facilitated sinking, but the latter provides greater volume capacity and more certainty of long-term sequestration. Both approaches may result in unexpected negative consequences, not least the potential for impact on deep sea ecosystems. Therefore, further research and thorough risk assessments of any proposed seaweed sinking activities (at scale) still need to be undertaken. In this nonharvest mariculture model, income would be generated through alternative funding streams such as payments through carbon markets, as opposed to markets based on harvested seaweed products. If 14% of current seaweed production (farmed within only 0.001% of the suitable area globally) were directed toward such carbon markets, this could offset all of the emissions from global aquaculture, enabling carbon neutrality for this industry (Froehlich et al. 2019).

A nonharvest carbon sequestration-based model for seaweed mariculture would necessitate large-scale operations and appropriate siting, likely in offshore areas (Pechsiri et al. 2016, Fernand et al. 2017). Such siting may also allow for proximity to deeper waters in which seaweed could be sunk and carbon sequestered (figure 5). However, the potential benefits of such offshore farms have to be weighed up against the additional transport and infrastructure related emissions from more distant farming operations. Ideal placement would likely be close to both deep waters and the coast (for maintenance and service access), meaning this industry may be most feasible in coastal waters in which the continental shelf is closest to the land and the biogeochemical and hydrodynamic environment supports productive seaweed growth and offshore transport of organic material.

If all the organic carbon from farmed seaweed was sequestered (i.e., not harvested) and the seaweed farming operations were carbon neutral; global seaweed mariculture could sequester between 0.05 and 0.29 Gt of CO2e per year (Hoegh-Guldberg et al. 2019). This is similar to the volume of carbon (0.16–0.25 Gt of CO2e per year) that could be sequestered through the restoration and protection of the world's mangroves (Hoegh-Guldberg et al. 2019). Although these seaweed carbon sequestration estimates could be viewed as a theoretical maximum for sequestration potential, they are arguably unrealistic, given the relatively limited current scale of seaweed mariculture. Therefore, attaining this amount of sequestration is unlikely in the short term. We are also not aware of any seaweed mariculture operations currently operating, at scale, under a carbon offsetting or trading model (although see Oceans 2050 2021, Running Tide 2021). Therefore, the realization of true carbon sequestration from this sector, although it is promising, remains an important area for action if ocean-based solutions to climate change through climate-friendly mariculture are to be realized (Hoegh-Guldberg et al. 2019).

### Is carbon sequestration achievable through bivalve mariculture?

The carbon embedded in bivalve shells during calcification equates to significant volumes when farmed at scale (e.g., an average of 0.67 (sd = 0.06) Mt of C stored in the shells of the 9.94 Mt of bivalves produced by Chinese mariculture in 2007; Tang et al. 2011), and there has been enthusiastic support for the potential of carbon sequestration from shells after harvesting (Hickey 2008). However, because bivalve shell formation and respiration are a net source of CO2 from sea to atmosphere (see above), the potential for bivalve monocultures to directly sequester carbon is limited (Munari et al. 2013, Filgueira et al. 2015). The cofarming of bivalves and seaweed can offset the carbon released by bivalves, which, under optimal conditions, can switch production from the net carbon source of bivalve monocultures, to a net carbon sink as bivalve–seaweed cocultures (Han et al. 2017). Seaweed primary production is usually carbon limited, but when grown close to bivalve mariculture, CO2 released by the bivalves can enhance seaweed photosynthesis. This in turn releases oxygen and improves conditions for bivalve cultivation (figure 5; Yang et al. 2005, Jiang et al. 2014), with an optimal ratio for carbon capture by seaweeds of 4:1 (bivalves to seaweed wet weight; Han et al. 2017). In China, where bivalve–seaweed cocultures are the primary form of bivalve mariculture, annual carbon removal is increased relative to bivalve monocultures by an average of 28.4% (sd = 0.1%; Tang et al. 2011). However, these mutual benefits are rarely integrated into bivalve mariculture outside of East Asia (Neori 2007). The labor, infrastructure and finance required to make such a change to cultivation practices and farm design could be a significant barrier, but, depending on the existing culture method and product markets, it may only require minor operational adjustments. For example, suspended bivalve farms could incorporate vertical seaweed cultures between bivalve stocks (e.g., baskets, line cultures), whereas the infrastructure for offshore bivalve cultures can readily incorporate seaweed longlines (Buck and Langan 2017). Of course, the capacity of such cocultures to truly sequester carbon (i.e., store it for hundreds to thousands of years) depends on the fate of the harvested bivalve shells (van der Schatte Olivier et al. 2020) and seaweed (Duarte et al. 2017), as was discussed in previous sections.

The various ecosystem regulating services associated with bivalve mariculture (Petersen et al. 2016, Alleway et al. 2019, van der Schatte Olivier et al. 2020) can influence the distribution and performance of blue carbon habitats, such as seagrasses, generating another potential source of indirect enhancement of marine carbon sequestration. Bivalve filter feeding on particulate matter encourages seagrass and macroalgal growth by reducing water turbidity, thereby increasing sunlight penetration (Wall et al. 2008, Humphries et al. 2016, Han et al. 2017). This filtering activity can also buffer coastal waters from eutrophication events by assimilating excess nutrients (Peterson and Heck 2001, Higgins et al. 2011, Willer and Aldridge 2020), some of which are deposited on the seabed as bivalve feces or pseudofeces, which acts as fertilizer and enhances seagrass growth (Reusch et al. 1994, Peterson and Heck 2001, Kent et al. 2017, Gagnon et al. 2020). However, in areas with particularly high nutrient loads, the additional fertilization of the benthic sediments by bivalves has been shown to limit the growth of seagrass (Wagner et al. 2012).

The influence of bivalve mariculture on blue carbon habitats, whether positive or negative, will be mediated by environmental setting, hydrodynamics and farm design (figure 1). Sheltered coastal systems that are slow flushing, shallow, and fringed by urban development are particularly vulnerable to nutrient enrichment (Ferreira and Bricker 2016). Such locations commonly support seagrass meadows and are suitable for bivalve mariculture, presenting opportunities to use bivalves as a cost-efficient means of extracting coastal nutrients through harvesting and, therefore, mitigating the impacts of eutrophication on blue carbon ecosystems (Newell 2004, Petersen et al. 2016, Theuerkauf et al. 2019). Maximizing the demonstrated potential of bivalve mariculture to cycle carbon and other nutrients to the seafloor (Sui et al. 2019) will require lease designs that encourage biodeposition and sediment stabilization, such as those that direct water over lease structures or away from the sediment surface to minimize resuspension (Comeau et al. 2014). Therefore, adopting mariculture designs that promote the regulating services and boost seagrass performance is necessary if bivalve mariculture is to indirectly enhance blue carbon sequestration (figure 5) as well as reducing potential for environmental GHG emissions.

### Is carbon sequestration achievable through fed finfish mariculture?

There is evidence of sediment accumulation and organic carbon enrichment under fed finfish mariculture net pens (figure 1; Carroll et al. 2003, Holmer et al. 2005, Tanner and Fernandes 2010, Yang et al. 2018), which is potentially significant in terms of sediment carbon sequestration (MacLeod et al. 2019). However, the spatial extent and magnitude of this process and whether it leads to meaningful increases in sediment organic carbon stocks are poorly studied and difficult to quantify (Henriksson et al. 2014a, 2014b). Some studies suggest that organic carbon accumulated in surface sediments under pens is highly labile, with increased carbon turnover rates (Liu et al. 2016, Eiríksson et al. 2017, Liu et al. 2017) and returns to baseline levels after fallowing (Lauer et al. 2009). This implies that organic carbon enrichment under sea pens is not a feasible mechanism for long-term carbon sequestration. In addition, the plethora of negative, ecological consequences associated with mariculture-derived sediment enrichment would likely negate any potential for emissions abatement benefits. These negative impacts include seagrass loss (as was described above) and the associated releases of blue carbon and other GHGs (López et al. 1998, Milewski 2001, Alongi et al. 2009, Liu et al. 2017, Moncada et al. 2019), oxygen depletion and anoxic sediments (Hargrave 2010), altered pore-water chemistry (Tanner and Fernandes 2010), a reduction in the abundance and diversity of benthic fauna, and a shift in the community composition of benthic microbes potentially increasing organic matter decomposition, a further release of GHGs and a breakdown of benthic–pelagic nutrient cycling (Weitzman 2019, Weitzman et al. 2019).

## Summary and recommendations

Although mariculture is typically reported as having a smaller GHG footprint than other food production industries (e.g., Hilborn et al. 2018, MacLeod et al. 2020), there are large differences in the median GHG emissions footprint of the fed finfish, bivalve and seaweed sectors and considerable variability within sectors (figures 2 and 3, table S1). This variation, and the critical need to rapidly reduce GHG emissions at a global level substantiate the need to identify and implement strategies that advance the climate-friendly capacity of mariculture, regardless of its current GHG emissions profile. The greatest opportunities for high-volume reductions in GHG emissions are likely to come from changes in upstream and downstream parts of the supply chain (figure 3; Hilborn et al. 2018). In particular, the method of postharvest transport has a large impact on the final GHG emissions footprint of maricultured products. High-value products (e.g., tuna and salmon) represent globally significant export industries reliant on rapid air freight to international markets. As the importation of seafood products becomes more viable in emerging economies there is the potential for GHG emissions associated with transport to increase substantially. Shortening supply chains and building regional markets could reduce GHG emissions at the same time, potentially contributing to greater food security (Belton et al. 2018) and industry resilience in times of crisis (Froehlich et al. 2021).

Because there is greater potential for mariculture farm operators to influence on-farm reductions in GHG emissions, we focused on on-farm activities and carbon exchange with the surrounding marine environment, aligning our climate-friendly assessment with the principles of designed industrial solutions (Francis 2016) and the FAO's Ecosystem Approach to Aquaculture, which seeks greater industry sustainability (Soto et al. 2007, Brugère et al. 2019). For mariculture to move decisively toward climate-friendly on-farm operations, we will need a concerted effort to reduce direct emissions (particularly through leveraging industry–environment interactions that alter local carbon flows; figure 1), and the development of market opportunities that support carbon sequestration and carbon offsetting. Unsurprisingly, there is not a single silver bullet solution that works in all sectors and situations. Rather, the sectors often have different interactions with the surrounding marine environment and operational practices that offer bespoke opportunities for either avoiding GHG emissions or enhancing carbon sequestration (figures 1, 4, and 5). Nevertheless, in synthesizing across the three sectors we identified six principles that can help enable climate-friendly mariculture approaches. These are theoretical and, while couched in the literature, they still require future research and development regarding cost–benefit analyses, detailed assessments of trade-offs with other environmental or socioeconomic factors, and investigations of the feasibility of scaling up.

### On-farm production can be emissions intensive

Climate friendly operations will need to find opportunities to increase efficiency (i.e., lower on-farm energy usage, shift to low-emissions energy sources, the use or reuse low-emissions, durable materials for farming infrastructure) and reduce nutrient inputs and wastes that lead to environmental GHG emissions, while also working toward carbon neutrality through the use of biofuels and clean energy sources to power on-farm operations.

### Interactions with surrounding marine environments influence GHG emissions

Our analysis highlights several farm design and operational changes that provide immediate options to reduce the impact of finfish and bivalve farming on benthic habitats and avoid environmental emissions of stored blue carbon (figures 4 and 5). These include siting fed finfish operations away from sensitive blue carbon habitats in deeper or faster flowing waters and minimizing feed waste to the environment (figure 4), as well as adopting climate-friendly grow-out strategies for bivalves that minimize benthic disturbance and sediment resuspension, such as using suspended bags and trays and manual harvesting methods (figure 5). There is also potential for both bivalve and seaweed mariculture to indirectly support or enhance blue carbon sequestration. For bivalves, this relies on positive interactions with surrounding marine environments that support sediment stabilization (and associated carbon burial), reduce eutrophication via filter feeding and increase benthopelagic coupling (i.e., transfer of fertilizing nutrients to the seafloor). Future research on the types of farm designs that promote the greatest biodeposition, sediment accumulation, and organic carbon burial (within ecological limits) is needed to understand the full potential for climate-friendly bivalve mariculture operations to deliver these benefits.

### Polyculture can support on-farm GHG emissions reductions

Opportunities for reducing indirect GHG releases from bivalve culture (i.e., the release of CO2 associated with calcification) and environmental degradation resulting from eutrophication around fed finfish farms may lie in polyculture approaches. These include cofarming bivalves with seaweed, which can lead to a net reduction in CO2 emissions (figure 5; Han et al. 2017) and cofarming fed finfish with seaweed or bivalves (Wang et al. 2014, Strand et al. 2019) that absorb excess nutrients, helping to reduce eutrophication and related blue carbon habitat degradation. However, the emissions abatement potential of such cross-sector synergies remains dependent on the fate of the farmed seaweed and bivalve shell waste, which would need to be repurposed through one of the potential applications that either enable ongoing carbon storage or offset emissions from other sectors (figure 5; Morris et al. 2019).

For carbon sequestration and GHG offsets, the fate of the product and scale of operation are key. There is a low immediate likelihood of long-term carbon sequestration from the sectors assessed. Seaweed mariculture holds the greatest potential for long-term carbon capture if seaweed is left in place (i.e., a carbon farming approach) and sequestration if transported to ocean carbon sinks. However, the potential negative impacts of these practices are unknown, and scaling up will be a challenge, because large quantities of biomass would need to be directed toward processes and markets that facilitate deep sea burial (Froehlich et al. 2019) or blue carbon donation (Trevathan-Tackett et al. 2015). Even if production was scaled up to account for 25% of total domestic aquaculture production tonnage in countries currently producing small volumes of seaweed, this would be unlikely to result in meaningful amounts of carbon sequestration (see the worked example in supplemental file S3 and table S5). Further barriers to the effective implementation and scaling of both seaweed and bivalve sequestration and offsetting strategies include a lack of recognition of seaweed carbon in current carbon accounting and trading markets, the potentially high costs of large-scale seaweed farming (particularly in offshore environments) compared with terrestrial carbon farming and the lack of large-volume markets for bivalve shell waste. To generate both scientific and market confidence in these proposed approaches, pilot projects designed to evaluate the potential sequestration and offsetting benefits, operational feasibility, and scale are needed (e.g., Oceans 2050 2021). These may include collecting data on on-farm nutrient fluxes, operational approaches to seaweed deep-sea transport and shell repurposing, potential for long-term sequestration, and full LCAs. Climate-friendly approaches will apply differently to different environmental and sectoral settings, and farms may need to pursue a portfolio of options to gain the greatest climate benefits while maintaining cost-effective operations.

### Thorough carbon accounting is critical

Unfortunately, variable methods and scope in mariculture GHG emissions LCAs mean that truly comparable studies accounting for the full supply chain are rare even for the same species and culture systems (Henriksson et al. 2013, Avadí et al. 2018, Bohnes et al. 2018, Bohnes and Laurent 2019). There is a clear need for more standardized reporting of GHG emissions to ensure comparability across studies within and between sectors, a challenge of LCAs that is not limited to the mariculture industry (Bohnes et al. 2018, Bohnes and Laurent 2019). Improvements here would provide a robust knowledge base with which to support mariculture's efforts to reduce and regulate its GHG footprint. In addition, the GHG emissions outcomes of both positive and negative interactions between mariculture operations and surrounding marine environments are missing from most LCA studies. We found that environmental emissions may contribute significantly to the industry's GHG footprint (supplemental material S2, table S4), therefore the true potential for avoided emissions from mariculture cannot be fully understood until these missing links are incorporated. Accurate LCAs for a range of exemplar species and environment combinations (as a starting point) may need to be done through industry audits that are incentivized by either (or both) regulation and climate-friendly certification. An increase in the availability of LCA tools and software that can be more easily used by a wide range of practitioners and that include appropriate options for the parameterization of mariculture operations (including environmental emissions) would improve the accuracy and feasibility of GHG accounting in mariculture.

### Availability of infrastructure, investment and value of end products may affect uptake of climate-friendly practices at farm, country, and regional scales

Some of the more innovative emissions reduction and sequestration practices suggested in the present article may need to be adopted early in regions with greater resources for research and development and greater capacity for investment in nascent technologies (Gentry et al. 2019).

## Conclusions

By linking the provision of food from mariculture to broader environmental benefits, such as GHG abatement, our study can support the development of climate-friendly mariculture practices that generate sustainable ecological, social, and economic outcomes (Tlusty et al. 2019). We also hope to assist in aligning the mariculture industry with carbon accounting, offsetting, and crediting schemes that are focused on either achieving demonstrable GHG emissions reduction or carbon sequestration. This is currently hindered by the lack of specific mariculture policy frameworks and knowledge gaps that prevent effective carbon accounting, particularly in the context of environmental (e.g., blue carbon) emissions and sequestration (Froehlich et al. 2019, Lovelock and Duarte 2019). Considering the projected global reliance on mariculture for food production into the future and the industry's persistently high growth rate (7.5% per year over the last 50 years; Bene et al. 2015, FAO 2020), sustainable intensification and the broadscale adoption of the Ecosystem Approach to Aquaculture will be critical to mitigating the climate impacts of a scaleup in mariculture production (Henriksson et al. 2018, MacLeod et al. 2019, Yuan et al. 2019, FAO 2020). This issue is particularly pertinent in this, the UN Decade of Ocean Science for Sustainable Development, which highlights the importance and opportunity for sustainable ocean provisioning to deliver equitable food and economic outcomes into the future (Bennett et al. 2019, Pretlove et al. 2019).

## Supplementary Material

biab126_Supplementary_Files

## Author Biographical

Alice Jones (alice.jones01@adelaide.edu.au), Dominic McAfee (dominic.mcafee@adelaide.edu.au), and Patrick Reis-Santos (patrick.santos@adelaide.edu.au) are marine ecologists at the University of Adelaide, in Adelaide, South Australia, in Australia. Heidi Alleway (heidi.alleway@tnc.org) is the global scientist, and Robert Jones (robert.jones@tnc.org) is the global lead for The Nature Conservancy's Aquaculture Program, in Arlington, Virginia, in the United States. Seth Theuerkauf (seth.theuerkauf@noaa.gov) is the science coordinator for the NOAA National Marine Fisheries Office of Aquaculture, in Silver Spring, Maryland, in the United States.
