# Supplementary material for: Climate-Friendly Seafood: The Potential for Emissions Reduction and Carbon Capture in Marine Aquaculture
Source: Bioscience. 2022 Jan 25;72(2):123–43. doi: 10.1093/biosci/biab126 (PMC8824708; doi:10.1093/biosci/biab126)
Supplement: biab126_Supplementary_Files [file biab126_supplementary_files.zip › Supplemental_File.docx]

**Supplementary material for Jones et al, 2021: *Climate-Friendly Seafood: The Potential for Emissions Reduction and Carbon Capture in Marine Aquaculture***

**S1: Data collation methods**

To lessen or mitigate mariculture related emissions and identify pathways to climate friendly practices, we first have to understand the associated greenhouse gas (GHG) emissions. We collated emissions data from the available literature to provide context around the GHG emission footprint from the three key mariculture sectors (fed-finfish, bivalves and seaweeds). This exercise aimed at investigating generalisable trends in emissions from on-farm/operational processes as well as related upstream and downstream processes, such that leverage points, key processes and opportunities for emissions abatement could be contextualised.

We searched for studies that met the following eligibility criteria: i) presented comparable information regarding system boundaries and ii) presented data on GHG emissions in (or convertible to) kg of CO_2_ equivalents (CO_2_e) per metric tonne (t) of harvested wet weight for marine seaweed, bivalve or finfish aquaculture production. We conducted a systematic literature review following the PRISMA (Preferred Reporting Items for Systematic Reviews and Meta-Analyses) guidelines (Moher et al. 2009). Boolean searches in Web of Science (June 2021) focused on the terms [aquaculture OR mariculture OR Finfish OR Bivalv* OR Seaweed] with combinations of the terms Impact*, Environment*, Greenhouse gas emission*, GHG, Carbon emission*, carbon footprint*, and life cycle assessment (see supplementary tables ‘Lit_review_metadata’ worksheet), where the asterisks act as a wildcard allowing all term derivatives to be discovered. These search terms resulted in a total of 798 unique studies (i.e., duplicates removed) that were investigated further to find comparable studies that quantitatively addressed GHG emissions from the three mariculture sectors (table S2). We first scanned the title and abstract of each study, eliminating 685 studies, with further eligibility checks of the body of text and supplements eliminating 76 studies that did not meet eligibility criteria. Overall, the main reasons for exclusion included studies not focusing on, or not quantifying emissions, or focusing on freshwater aquaculture. In the end, a total of 38 studies (published between 2006 and 2021) were compiled. We then reviewed the reference lists of the selected studies for additional articles or reports that may contain useable data, and performed equivalent (Google) searches targeting technical reports from industry, government and research, development and extension agencies. Whilst no additional peer reviewed studies were found, 12 reports with emissions data were identified and added to our list (Total N = 50). Each selected study was given a Study ID and the basic bibliographic information recorded (table S1).

Of the 50 identified studies, 8 contained data for seaweed, 14 for bivalves, and 28 for Finfish (table S1). Data were taken directly from the text or published tables. Where this was not possible, data were extracted from figures using Webplotdigitizer (Rohatgi 2015). Overall, the main sources of information were LCA studies and technical reports on mariculture carbon emissions, as well as the Seafood Carbon Emissions website (for fed-finfish; Max et al. 2020). The data were sometimes reported in units that required manual back-calculation from emissions expressed in other functional units (e.g., emissions per volume of biofuel produced; this being the downstream production system most commonly reported in the seaweed literature). The information on GHG emissions extracted from individual papers were characterised by whether they were associated with on-farm (e.g. operational aspects such as fuel use, coastal and sub-tidal land-use changes), upstream (e.g. egg/larvae supply, feed production) or downstream processes (e.g. processing, packaging, transport to market; table S1). If papers contained data on multiple species, or explored different scenarios regarding emissions at any stage, these were recorded as different entries in the data table (table S1). The final dataset used in this study is provided in table S3.

All supplementary tables are available to download from the following link: <https://figshare.com/s/7644ccb14a75fa9d8c82>

**S2: Worked example of potential for GHG emissions from loss of seagrass around fed-finfish mariculture net pens**.

Halwart et al (2007) and papers therein provide estimates of coastal mariculture net pen footprints for specific regions and countries between 2000 and 2004 (Thailand, Malaysia, Indonesia, Hong Kong, Chile, Mexico, Canada and Slovenia), which total 375.6 thousand hectares (3756 km^2^; table S4). Unfortunately, there are no georeferenced global data available with which to estimate the spatial distribution of active fed-finfish mariculture net pens, nor their overlap with seagrass habitats. However, seagrasses generally thrive in both tropical and temperate settings in the same shallow, protected areas that are most favourable for net pen mariculture (Short et al. 2007), and therefore the overlap in these regions may be considerable. There is also clear evidence from multiple global regions that the negative impacts of fed-finfish mariculture can extend a considerable distance beyond the footprint of the net pens (Carroll et al. 2003, Díaz-Almela et al. 2008, Henderson et al. 2001). Therefore, we have explored the potential for seagrass and associated blue carbon losses based on a range of 10 % (37,561 ha/375.6 km^2^) to 40 % (150,247 ha/1502 km^2^) overlap between net pens and seagrass habitats. We assume that there is complete seagrass loss within the overlapping area and that this causes:

- The complete loss of all blue carbon stored in the seagrass *biomass*, which is estimated to be 9.2 t CO_2_e per ha based on the global average estimate from Fourqurean et al (2012); and
- The partial (50 %) loss of blue carbon stored in the top 1-m of sediment, which is estimated to be 512.7 t CO_2_e per ha based on global estimates from Fourqurean et al, (2012). Noting that this is a conservative scenario, as Pendleton et al (2012) suggest that up to 100 % of the near-surface sediment carbon may be lost to the atmosphere under some seagrass loss scenarios.

According to these overlap and carbon loss scenarios, the potential release of stored blue carbon (from both biomass and sediment carbon stocks) could range from 9.98 million t CO_2_e (10 % overlap) to 39.9 million t CO_2_e (40 % overlap), which is equivalent to between 4.1 – 12.2 % of the emissions from all aquaculture (both freshwater and marine) in 2017 (245 million t CO2e; MacLeod et al. 2020). This loss of seagrass would also prevent the future sequestration of between 190.2 and 760.9 thousand t CO_2_e per year going forward (depending on the overlap scenario), based on a global average seagrass carbon sequestration rate of 5.1 t CO_2_-e per ha (Mcleod et al. 2011; see calculations in table S4). This loss of ongoing yearly sequestration into seagrasses would equate to between 0.08 and 0.31 % of the annual GHG emissions from aquaculture in 2017 (MacLeod et al. 2020).

Clearly these estimates (table S4) are based on broad assumptions about the overlap between coastal net pens and seagrasses, the severity of the impact of sea pens on seagrasses and their blue carbon stocks, and an absence of effective regulations restricting marine aquaculture from being sited in and around seagrasses. However, the sea pen footprint we have used is based on just a fraction of the countries producing fed-finfish mariculture (i.e. only those where spatial data are available), with a more recent study estimating a global net pen footprint of 23,000 km^2^or 2.3 million ha (Bugnot et al. 2021). Notably, our area estimates do not cover China (the largest fed-finfish mariculture producer, by far) or any large, salmon producing nations (such as Norway and Chile), and they are more than 15 years out of date for a rapidly growing aquaculture sector (FAO 2020, Halwart et al. 2007). Therefore, we consider it likely that the current releases of stored blue carbon and losses of ongoing sequestration from seagrass, due to fed-finfish mariculture operations, are far greater than the estimate given above. There is clearly a need for consistent, georeferenced data on the global area coverage and spatial distribution of coastal net pens, and their overlap with sensitive benthic habitats. This would enable accurate accounting of this potentially large source of environment GHG emissions (Pendleton et al. 2012).

**S3: Worked example of scaling up global seaweed mariculture production for carbon sequestration benefits**.

The value of seaweed mariculture in facilitating carbon sequestration through non-harvest farming approaches (e.g. Froehlich et al. 2019) is predicated on the high rate at which production in this sector is increasing and the generally high productivity rates of seaweed farming, with the potential for fast growth and intra-annual harvest. Of the top 20 mariculture producers in 2018, only 12 registered production for marine aquatic plants. Production of seaweeds in China, Indonesia, the Democratic People’s Republic of Korea (DPRK), Philippines, Japan, Democratic People’s Republic of Korea, and Malaysia, comprised more than 38 % of each country’s total aquaculture production (table S5). But in the remaining seaweed-producing countries (Vietnam, Norway, Chile, India, India, Ecuador, Thailand, Spain, Egypt, Bangladesh, Turkey, USA, UK, and Canada), production quantities comprised less than 1.7 % of total production from each country (table S5). If each of these low-producing nations were to increase their capacity to produce marine plants to mirror the proportions of the DPRK, Indonesia, the Philippines etc. (conservatively targeted at 25 % of total marine aquaculture production), the total quantities of seaweed produced across all nations could potentially capture over 3.7 million t CO_2_e yr^-1^ (table S5). This value is based on a 10:1 wet weight to dry weight conversion of total aquatic plant tonnage, and an estimated average organic carbon content of 30 % and subsequent multiplier of 3.67 to convert from organic carbon to CO_2_ equivalents. For this stored carbon to be truly sequestered, the seaweed would need to be directed towards a non-harvest market (e.g. carbon crediting) or used to make products that offset emissions from other sources such as biofuels (Duarte et al. 2017, Sondak et al. 2017). This is a significant assumption and limitation to seaweed mariculture’s current value in realising an effective carbon mitigation strategy.

This potential sequestration value, based on scaling up seaweed production, represents 1.5 % of the recently-estimated 245 million t CO_2_e in emissions from all forms of aquaculture (inland and marine) in 2017 (MacLeod et al. 2020), and 0.05 % of the 7.1 billion t CO_2_e yr^-1^ from global livestock production (Ritchie 2019). If seaweed production was able to be scaled up to reach 25 % of total aquaculture production in the low-producing countries (and all carbon was sequestered), this would only offset a maximum of 0.1 % of domestic GHG emissions in any of those countries (0.1 % in Norway, followed by 0.04 % in Chile; table S5). The effort and expenditure required to ‘activate’ this scale of development is substantial; investment that must be weighed against the relative value for carbon sequestration as a country-scale option for CO_2_ mitigation.

**References:**

Bugnot AB, et al. 2021. Current and projected global extent of marine built structures. Nature Sustainability 4:33-41.

Carroll ML, Cochrane S, Fieler R, Velvin R, White P. 2003. Organic enrichment of sediments from salmon farming in Norway: environmental factors, management practices, and monitoring techniques. Aquaculture 226:165-180.

Díaz-Almela E, et al. 2008. Benthic input rates predict seagrass (Posidonia oceanica) fish farm-induced decline. Marine Pollution Bulletin 56:1332-1342.

Duarte CM, Wu J, Xiao X, Bruhn A, Krause-Jensen D. 2017. Can seaweed farming play a role in climate change mitigation and adaptation? Frontiers in Marine Science 4:100.

FAO. 2020. The State of the World Fisheries and Aquaculture - Sustainability in Action. Rome: Food and Agriculture Organisation. Report no.

Fourqurean JW, et al. 2012. Seagrass ecosystems as a globally significant carbon stock. Nature Geoscience 5:505-509.

Froehlich HE, Afflerbach JC, Frazier M, Halpern BS. 2019. Blue Growth Potential to Mitigate Climate Change through Seaweed Offsetting. Current Biology 29:3087-3093.e3083.

Halwart M, Soto D, Arthur JR. 2007. Cage Aquaculture - Regional Reviews and Global Overview. Rome, Italy: Food and Agriculture Organisation. Report no. FAO Fisheries Technical Paper 498.

Henderson, Gamito, Karakassis, Pederson, Smaal. 2001. Use of hydrodynamic and benthic models for managing environmental impacts of marine aquaculture. Journal of Applied Ichthyology 17:163-172.

MacLeod M, Hasan MR, Robb DHF, Mamun-Ur-Rashid M. 2020. Quantifying greenhouse gas emissions from global aquaculture. Scientific reports 10:11679.

Max L, Parker R, Tyedmers P. 2020. Seafood Carbon Emissions Tool,. (19/01/2021 2021; http://seafoodco2.dal.ca/)

Mcleod E, Chmura GL, Bouillon S, Salm R, Björk M, Duarte CM, Lovelock CE, Schlesinger WH, Silliman BR. 2011. A blueprint for blue carbon: toward an improved understanding of the role of vegetated coastal habitats in sequestering CO2. Frontiers in Ecology and the Environment 9:552-560.

Moher D, Liberati A, Tetzlaff J, Altman DG. 2009. Preferred reporting items for systematic reviews and meta-analyses: the PRISMA statement. BMJ 339:b2535.

Pendleton L, et al. 2012. Estimating Global “Blue Carbon” Emissions from Conversion and Degradation of Vegetated Coastal Ecosystems. PLOS ONE 7:e43542.

Ritchie H. 2019. Food production is responsible for one-quarter of the world’s greenhouse gas emissions. (18/2/2021 2021; https://ourworldindata.org/food-ghg-emissions)

Rohatgi A. 2015. Webplotdigitizer. (2021; https://automeris.io/WebPlotDigitizer)

Short F, Carruthers T, Dennison W, Waycott M. 2007. Global seagrass distribution and diversity: A bioregional model. Journal of Experimental Marine Biology and Ecology 350:3-20.

Sondak CFA, et al. 2017. Carbon dioxide mitigation potential of seaweed aquaculture beds (SABs). Journal of Applied Phycology 29:2363-2373.
